# Schistosomiasis Screening of Travelers to Corsica, France

**DOI:** 10.3201/eid2201.151290

**Published:** 2016-01

**Authors:** Antoine Berry, Luc Paris, Jérôme Boissier, Eric Caumes

**Affiliations:** Toulouse University Hospital, Toulouse, France (A. Berry); Public Assistance Hospitals of Paris, Paris, France (L. Paris, E. Caumes);; University of Perpignan Via Domitia, Perpignan, France; National Centre of Scientific Research, Perpignan (J. Boissier)

**Keywords:** schistosomiasis, screening, travelers, exposure, surveillance, Schistosoma, haematobium, mansoni, parasites, trematode, snail, intermediate host, urinary schistosomiasis, Bulinus truncatus snail, One Health, freshwater rivers, SCHISTO II WB IgG test, Western blot, Cavu River, Corsica, France, Italy, Europe

**To the editor:** As members of the French Ministry of Health Working Group on autochthonous urinary schistosomiasis, we read with interest the 2 recently published articles regarding schistosomiasis screening of travelers to Corsica, France (*1*,*2*). Surprisingly, the authors of both articles lacked evidence to support the diagnosis of schistosomiasis in most of what they referred to as confirmed cases. The diagnostic standard for confirmation of urinary schistosomiasis is identification of eggs by microscopic examination of urine samples (*3*–*5*). If this criterion were applied in both reports, only 1 patient of the 7 allegedly confirmed cases would actually be confirmed.

The low sensitivity of microscopy is well known. Therefore, different serologic tests have been developed, including Western blot (WB). In the study based on travelers from Italy (*1*), the SCHISTO II WB IgG test (LDBIO Diagnostics, Lyon, France) was used. This test, available since 2015, is based on both *S*c*histosoma haematobium* and *S. mansoni* antigens and has not been evaluated by anyone other than the manufacturer. Moreover, the authors did not report any details regarding the molecular weight and number of specific bands observed on the strip.

In the study by authors from the GeoSentinel Surveillance Network (*2*), both cases that could have been infected after 2013, since exposure occurred only in 2014, and 4 cases which reported bathing in rivers in Corsica other than the Cavu River had just 1 weakly positive serologic screening test. Hence, irrespective of the criteria for a confirmed case of schistosomiasis described above, it appears difficult to conclude that confirmation could rely on only 1 positive serologic test, even a WB.

Altogether, these 2 studies identified only 1 patient with parasitological evidence of infection that was attributable to the already known 2013 focus in Cavu River. Therefore, these articles do not provide evidence of transmission of schistosomiasis in Corsica after 2013 or outside the Cavu River.
